# Case report: Complex paraneoplastic syndromes in thymoma with nephrotic syndrome, cutaneous amyloidosis, myasthenia gravis, and Morvan’s syndrome

**DOI:** 10.3389/fonc.2022.1002808

**Published:** 2022-11-21

**Authors:** Huiqin Liu, Zeqin Dong, Milan Zhang, Rui Pang, Jiajia Xu, Pan He, Wenli Mei, Shuai Zhang, Guanqiao You, Wei Li

**Affiliations:** ^1^ Department of Neurology, Henan Provincial People’s Hospital, Zhengzhou, China; ^2^ Department of Nephrology, Henan Provincial People’s Hospital, Zhengzhou, China; ^3^ Department of Electromyography, Henan Provincial People’s Hospital, Zhengzhou, China; ^4^ Department of Dermatology, Henan Provincial People’s Hospital, Zhengzhou, China

**Keywords:** thymoma, myasthenia gravis, membranous nephropathy, cutaneous amyloidosis, morvan’s syndrome, paraneoplastic syndromes, leucine-rich glioma inactivated 1

## Abstract

**Background:**

Apart from myasthenia gravis (MG), thymoma is associated with a wide spectrum of autoimmune paraneoplastic syndromes (PNSs). Here, we report on a rare case presenting with four different PNSs, namely, MG, membranous nephropathy, cutaneous amyloidosis, and Morvan’s syndrome associated with thymoma.

**Case presentation:**

A middle-aged man was frequently hospitalized because of nephrotic syndrome (stage I membranous nephropathy), cutaneous amyloidosis, and MG with acetylcholine receptor (AChR) antibody and titin antibody positivity. Chest CT showed a thymic mass in the left anterior mediastinum, and he received intravenous immunoglobulin (IVIG), methylprednisolone pulse therapy, thoracoscopic thymoma resection, and radiotherapy. Postoperative pathological examination revealed a type B2 thymoma. During the perioperative stage, his electrocardiogram (ECG) showed myocardial infarction-like ECG changes; however, his levels of cardiac enzymes and troponin were normal, and he had no symptoms of precardiac discomfort. Six months after thymectomy, his nephrotic syndrome and MG symptoms were relieved; however, he presented with typical manifestations of Morvan’s syndrome, including neuromyotonia, severe insomnia, abnormal ECG activity, and antibodies against leucine-rich glioma-inactivated 1 (LGI1) and γ-amino-butyric acid-B receptor (GABABR). His symptoms did not improve after repeated IVIG and steroid therapies. Finally, he received low-dose rituximab, and his symptoms gradually resolved.

**Conclusion:**

This case serves to remind us that apart from MG, thymoma is also associated with other autoimmune PNSs such as membranous nephropathy, cutaneous amyloidosis, and Morvan’s syndrome. Autoimmune PNSs can present concurrently with or after surgical or medical therapy for thymoma. For Morvan’s syndrome post-thymectomy with LGI1 antibody positivity, B-cell depletion therapy such as intravenous rituximab is an effective treatment.

## Introduction

Apart from myasthenia gravis (MG), thymoma is also associated with a wide spectrum of autoimmune paraneoplastic syndromes (PNSs), including systemic lupus erythematosus, autoimmune cytopenia, neuromyotonia, Morvan’s syndrome, limbic encephalitis, polymyositis, Good’s syndrome, autoimmune thyroid diseases, autoimmune hepatitis, and cutaneous autoimmune disorders (1–6). The clinical manifestations of PNSs associated with thymomas pose a challenge to clinicians because of the need to decipher the association between the presenting symptoms and the underlying mass. Here, we report a rare case of four different PNSs associated with thymoma: nephrotic syndrome (stage I membranous nephropathy), cutaneous amyloidosis, MG with acetylcholine receptor (AChR) and titin antibodies, and Morvan’s syndrome post-thymectomy with antibodies against leucine-rich glioma-inactivated 1 (LGI1) and γ-amino-butyric acid-B receptor (GABABR). The symptoms of nephrotic syndrome, cutaneous amyloidosis, and MG gradually improved after receiving intravenous immunoglobulin (IVIG), steroids, thoracoscopic thymoma resection, and radiotherapy. The symptoms of Morvan’s syndrome post-thymectomy did not improve after receiving IVIG and oral steroids but were relieved after receiving intravenous rituximab therapy.

## Case presentation

**Figure 1:**
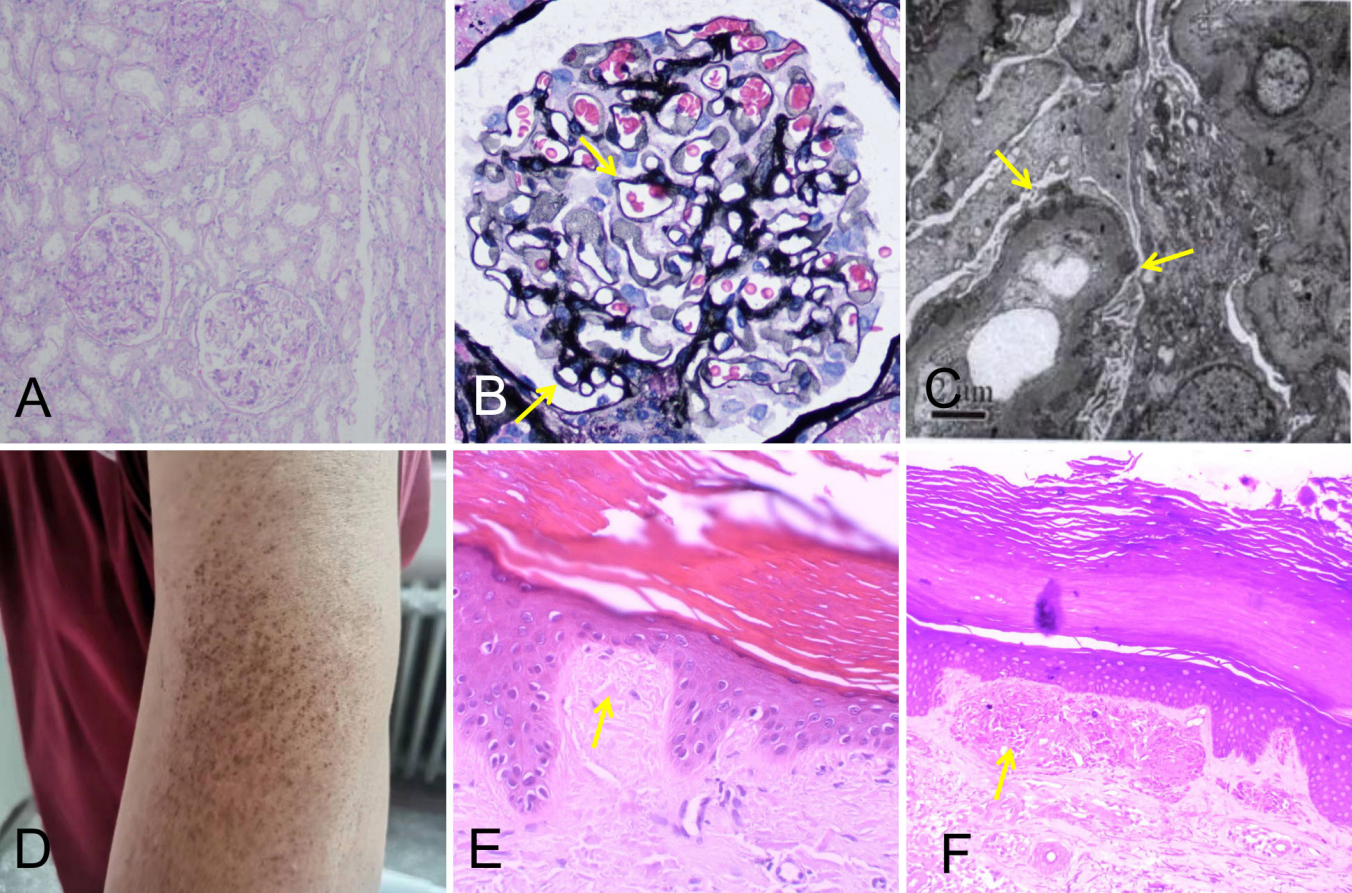
Kidney biopsy findings showing stage I membranous nephropathy **(A–C)**. **(A)** Normal mesangial cellularity (periodic acid–Schiff; original magnification, ×100). **(B)** Slight global glomerular basement membrane thickening (yellow arrows; Jones methenamine silver; original magnification, ×400). **(C)** Subepithelial electron-dense deposits, partially in the glomerular basement membrane (yellow arrows; original magnification, ×4,000). **(D)** Skin lesions on the patient’s left upper arm. **(E, F)** Skin biopsy findings showing cutaneous amyloidosis. **(E)** The skin specimen stained by hematoxylin–eosin revealed parakeratosis with focal hyperkeratosis, widened dermal papilla, and an amorphous red-stained mass substance (yellow arrow; ×400). **(F)** The skin specimen stained by crystal violet was positive (yellow arrow; ×400).

A 49-year-old male patient was admitted to the Department of Nephrology of Henan Provincial People’s Hospital on 23 March 2020. He presented with symptoms of edema of both lower limbs for 2 months and eyelid edema for 15 days. Physical examination showed pitting edema of both legs, edema of double eyelids, and extensive pigmentation of the skin of the whole body. The patient subsequently underwent a series of examinations. The results showed that his level of 24-h urinary protein was 21.72 g/L, level of serum albumin was 9.5 g/L, serum total cholesterol was 9.07 mmol/L, creatinine was 125 μmol/L, and titer of antinuclear antibody (ANA) was 1:1,000, and he was also positive for anti-histone antibody (AHA). His electrocardiogram (ECG) at that time was normal. This patient underwent kidney biopsy, and the biopsy revealed that there was mild thickening of the glomerular basement membrane, normal mesangial cellularity, and no interstitial fibrosis or tubular atrophy. Immunofluorescence staining showed granular deposits along the capillary walls for IgG, C3, kappa, and lambda light chains, and electron microscopy showed subepithelial electron-dense deposits. Based on the above observations, the patient was diagnosed with stage I membranous nephropathy (**Figure 1**). This patient also underwent skin biopsy, where pathology showed cutaneous amyloidosis (**Figure 1**). He received methylprednisolone at an initial dosage of 40 mg per day and intravenous cyclophosphamide at a total dose of 2 g, with steroids tapered down gradually. In addition, he received treatment such as intravenous albumin supplementation and diuresis.

**Figure 2:**
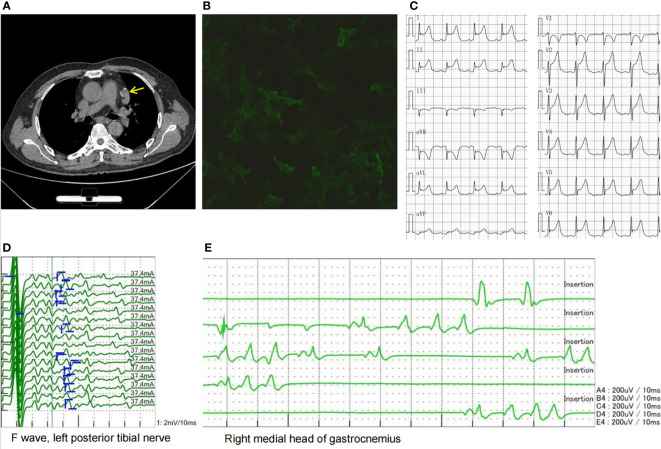
**(A)** Chest CT showing a thymic mass in the left anterior mediastinum (yellow arrow). **(B)** LGI1 antibody cell-based assay showing a 1:32 positive ratio. **(C)** ECG showing acute anterior, lateral, and high lateral myocardial infarction-like ECG changes. **(D)** Afterdischarges were observed in the F wave. **(E)** Needle EMG showing duplet, triplet, and multiplet bursts of spontaneous motor unit discharges in the tested muscles of the limbs at rest suggestive of myokymia. CT, computed tomography; LGI1, leucine-rich glioma-inactivated 1; ECG, electrocardiogram; EMG, electromyography.

Four months after the above examinations, the patient was rehospitalized in our hospital for follow-up on 10 August 2020. The edema of his legs and eyelids had disappeared, the concentration of 24-h urinary protein decreased to 0.77 g/L, and the serum creatinine level had returned to normal. Thus, the steroid dosage was tapered to 20 mg/day. However, the patient complained of weakness in his neck and both hands, as well as chest distress, shortness of breath, inability to lie flat, and dysphagia. These symptoms were indicative of the fatigue phenomenon. Subsequent neostigmine test results were positive, while repetitive nerve stimulation showed 51.4% and 31% decremental compound muscle action potential responses at low- and high-stimulation frequencies, respectively. The patient underwent chest computed tomography (CT), which revealed a thymic mass in the left anterior mediastinum (**Figure 2**). Antibodies associated with MG in his serum were tested and demonstrated the following: the level of AChR antibody was higher than 320 nmol/L, and his titin antibody was also strongly positive.

The patient was diagnosed with thymoma-associated MG based on the observations above and received treatments such as IVIG at a dose of 0.4 g/kg/day * 5 days, methylprednisolone pulse therapy, and pyridostigmine administration. His muscle weakness then gradually improved. Subsequently, the patient underwent thoracoscopic thymoma resection using the subxiphoid approach under general anesthesia. The size of the thymus tissue examined was 5 × 3 * 1.5 cm, and its pathological diagnosis was type B2 thymoma. After surgery, the patient was transferred to the intensive care unit for respiratory support, anti-infection treatment, and immunomodulatory treatment. During this period, his ECG showed acute anterior, lateral, and high lateral myocardial infarctions (**Figure 2**); however, the myocardial enzyme profile and troponin levels were normal, and he had no symptoms of precardiac discomfort. After a period of recovery, he was transferred to the oncology department for radiotherapy. The patient was discharged on 28 November 2020. At that time, his symptoms of MG were relieved, the level of AChR antibody decreased to 19.6 nmol/L, and he was able to take care of himself.

**Figure 3:**
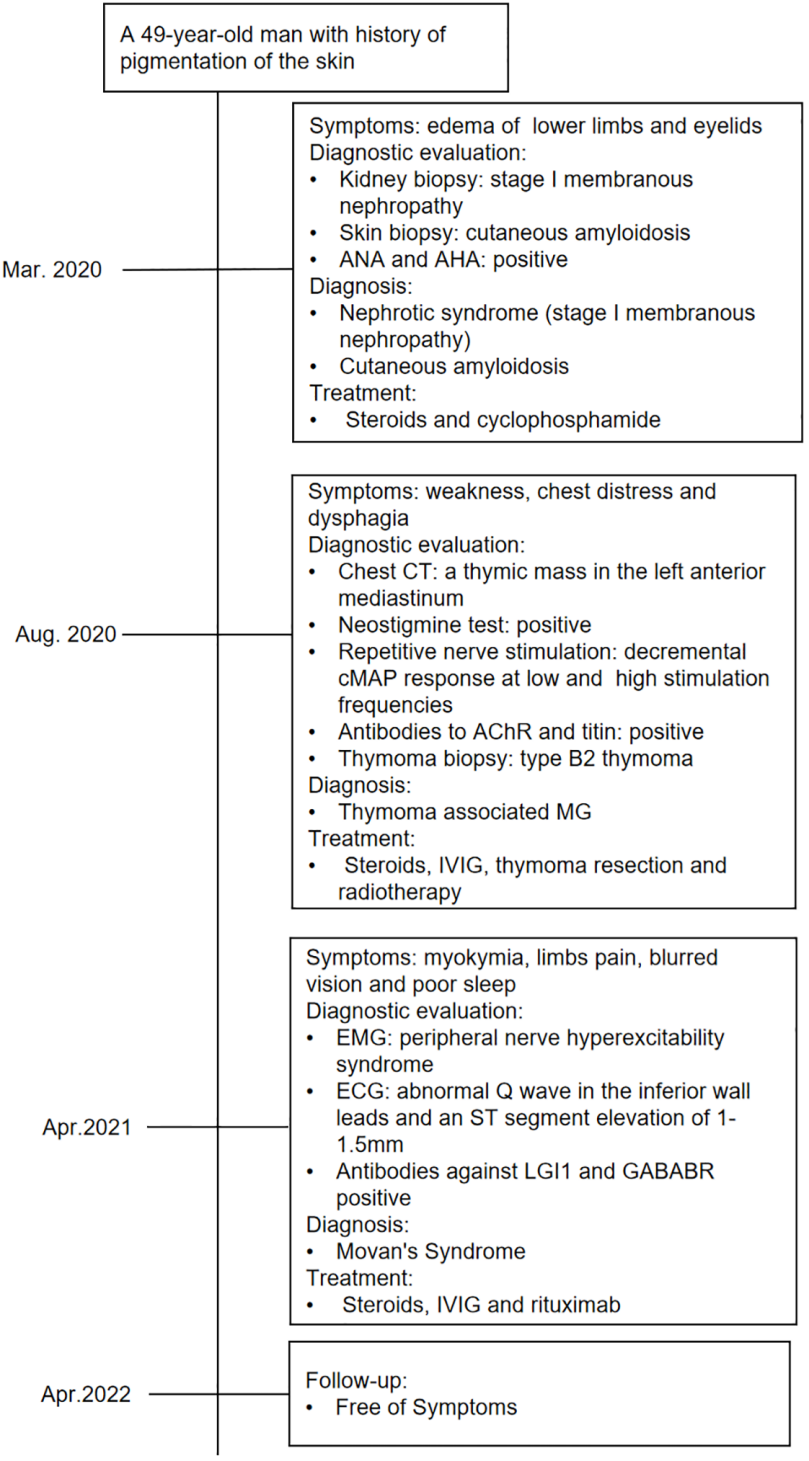
Timeline of the diagnosis, treatment, and outcome. CT, computed tomography; cMAP, compound muscle action potential responses.

In the following 6 months, the patient received oral prednisone and pyridostigmine bromide with regular follow-up. On 15 April 2021, the patient was readmitted for follow-up. His nephrotic syndrome and MG symptoms were relieved with 10 mg of oral prednisone per day. However, he complained of diffuse muscle twitching within the last week, as well as limb pain, blurred vision, and poor sleep. He could only sleep for 1–2 h every night. Physical examination revealed extensive myokymia in the limbs and trunk involving spontaneous, continuous, undulated muscle movements, similar to a bag of worms under the skin (**Video 1**). Needle electromyography (EMG) showed duplet, triplet, and multiplet bursts of spontaneous motor unit discharges in the tested muscles of the limbs at rest, and after discharges were observed in the F wave, suggesting peripheral nerve hyperexcitability syndrome (**Figure 2**). His ECG showed abnormal Q waves in the inferior wall leads and an ST-segment elevation of 1–1.5 mm. Blood tests showed that apart from ANA and AHA, anti-neutrophil cytoplasmic antibody (ANCA) was also positive. The patient’s serum was also tested for antibodies to cell-surface antigens, including N-methyl-D-aspartate receptor, LGI1, contactin-associated protein 2 (CASPR2), GABABR, and alpha-amino-3-hydroxy-5-methyl-4-iso-xazolepropionic acid (AMPA) receptors (EUROIMMUN, Germany), and the results showed that antibodies against LGI1 and GABABR were both positive, with titers of 1:32 (**Figure 2**) and 1:10, respectively. The patient was diagnosed with Morvan’s syndrome and treated with IVIG at a dose of 0.4 g/kg/day for 5 days and oral steroids; however, his symptoms of myokymia and insomnia did not improve 1 month later. Considering that the patient’s immune test indicated that he was in an immunosuppressive state at the time, he was treated with low-dose rituximab (7, 8) at a dose of 100 mg/week for 4 weeks. During the follow-up 1 month later, the muscle twitching, limb pain, and insomnia symptoms were significantly improved. Subsequently, the patient received 100 mg of rituximab intravenously every 6 months, and his symptoms have not recurred for 1 year. The timeline of the diagnosis and treatment is shown in **Figure 3**.

## Discussion

Thymomas are epithelial tumors arising from the thymus and are the most commonly found tumors in the anterior mediastinum. Approximately 40%–50% of thymomas present with PNSs (5, 9). It has been noted that 25%–40% of patients with thymoma present with MG, and more than 15% of patients diagnosed with thymoma present with a PNS other than MG (2, 4, 10, 11). PNSs can be antibody- or non-antibody-mediated, leading to both organ-specific and systemic effects (2, 4, 11). Approximately one-third of patients with PNSs have two or more conditions (9). PNSs can also present before, concurrently with, or after surgical or medical therapy for thymomas (5, 11). Here, we present a rare case of a middle-aged male patient who was successively diagnosed with multiple thymoma-associated PNSs, including membranous nephropathy, cutaneous amyloidosis, MG, and Morvan’s syndrome, as well as with multiple positive serum antibodies (ANA, AHA, ANCA, AChR, titin, LGI1, and GABARB).

This patient was diagnosed with membranous nephropathy at the first visit due to nephrotic syndrome, and his symptoms were completely relieved after receiving steroids, cyclophosphamide, and thymectomy. Thymoma-associated nephropathy is very rare, with only 10% of nephrologists in France encountering patients with thymoma-associated renal disease (12). In thymoma-associated nephropathy, the most common pathology is minimal change glomerulopathy, followed by membranous nephropathy, focal segmental glomerulosclerosis, and others (12). Membranous nephropathy is usually associated with active thymoma, either newly diagnosed or recurrent. Tumor treatment (thymectomy, radiotherapy, or chemotherapy) frequently induces remission of nephrotic syndrome in membranous nephropathy (12). The good outcome in this patient confirmed this view. Immunological tests were disturbed in many cases of thymoma-associated nephropathy, and it was reported that ANA was positive in 13/18 (72%) of such cases (12). Our patient also showed many autoantibodies, such as ANA, AHA, and ANCA.

Cutaneous disorders associated with thymomas are widely heterogeneous and include pemphigus, lichen planus, vitiligo, alopecia areata, lupus erythematosus, and graft-versus-host-like disease (5, 13). However, our case is the first report of cutaneous amyloidosis associated with thymoma. As previously reported, the effect of thymectomy seems to be variable but can, in some cases, induce the regression of paraneoplastic cutaneous disorders (14, 15). In view of this, the patient’s skin manifestations improved after complete thymoma resection.

MG is the most common PNS associated with thymoma, with 10%–15% of MG patients presenting with thymoma and 25%–40% of patients with thymoma developing MG (2, 4, 10). Nearly all patients with thymoma-associated MG have detectable AChR antibodies (1, 10, 16). The AChR antibody concentration in our patient was very high, reaching 320 nmol/L. To date, no correlation has been observed between AChR antibody concentration and disease severity. The value of repeated AChR antibody testing in patients with this disorder has been debated; however, changes in antibody concentrations may predict disease severity in patients administered immunosuppressive drugs and can therefore support therapeutic decisions (16). After our patient underwent thymoma resection and immunotherapy, the concentration of AChR antibody decreased to 19.6 nmol/L, supporting the view that longitudinal observation of AChR antibody concentrations is needed for the long-term treatment of MG patients. In addition, the titer of the titin antibody in this patient was very high. The presence of titin antibody is suggested to be a strong indication of thymoma and is associated with more severe MG (17).

Acquired neuromyotonia (aNMT) is a disorder characterized by peripheral nerve hyperexcitability that results in continuous muscle activity. Clinical presentations include myokymia, fasciculations, and cramps (3, 18), and EMG shows spontaneous motor unit discharges as doublet, triplet, or multiplet bursts (myokymic discharges) or longer bursts with high intraburst frequencies (neuromyotonic discharges) (18). The clinical symptoms and EMG findings of this patient were typical manifestations of neuromyotonia. In 20% of cases, aNMT is associated with signs of central nervous system involvement, such as mood changes, hallucinations, and insomnia, together with autonomic dysfunction, collectively known as Morvan’s syndrome (19). Aside from neuromyotonia, the patient’s symptoms of severe insomnia, blurred vision, and myocardial infarction-like ECG changes support the diagnosis of Morvan’s syndrome.

Morvan’s syndrome is a rare disease associated with thymoma. In a study of 29 patients with Morvan’s syndrome, thymoma was found in 37% of the patients (19). aNMT is associated with antibodies to the Kv1 voltage-gated potassium channel complex. These antibodies do not target Kv1 channels directly; rather, they target two associated proteins, LGI1 and CASPR2 (6, 20, 21). In this patient, antibodies against LGI1 and GABABR were both positive, while antibodies against CASPR2 were negative. The LGI1 antibody was pathogenic in this patient, as it has been reported to alter Kv1.1 and AMPA receptors and modify synaptic excitability, plasticity, and memory (22). In addition, the role of the GABABR antibody in this patient’s disease is not clear, and it may only be a concomitant antibody.

The ECG of the patient showed serious abnormalities similar to those of myocardial infarction. A similar phenomenon has been reported in previous literature: severe bradycardia and even sudden death due to myocardial ischemia with normal coronary arteries occurred in patients with LGI1 antibody-associated encephalitis (21, 23). This emphasizes that in patients with Morvan’s syndrome associated with LGI1 antibody, cardiac complications are potentially life-threatening and require clinical vigilance. The patient received IVIG therapy after he was diagnosed with Morvan’s syndrome; however, his symptoms did not improve. The reason why IVIG did not work might be that most subclasses of LGI1 antibodies belong to IgG4, which is inadequate for activating a cellular- or complement-mediated immune response (24). The patient recovered after receiving B-cell depletion therapy. His good response to rituximab is consistent with previous research that reported the effectiveness of rituximab treatment in patients with LGI1 encephalitis (25).

We questioned why multiple coexisting autoimmune syndromes occur in patients with thymoma. Notably, the thymus is a key site for the establishment of immune tolerance. This process involves the maturation and selection of T cells during their migration through the thymic cortex and medulla. Thus, defective negative selection with the export of autoreactive CD4^+^ T cells together with a reduced level of regulatory T cells may appear to be the key features associated with the occurrence of PNS in thymoma patients (4, 26).

## Conclusion

Overall, we present a rare case of a middle-aged male patient who was successively diagnosed with multiple thymoma-associated PNSs, including membranous nephropathy, cutaneous amyloidosis, MG, and Morvan’s syndrome, and who presented with multiple positive serum antibodies (ANA, AHA, ANCA, AChR, titin, LGI1, and GABARB). This case serves to remind us that, apart from MG, thymoma may also be associated with other autoimmune PNSs. Thymectomy, related tumor therapy, and immunotherapy are important for the management of PNSs. For Morvan’s syndrome post-thymectomy with LGI1 antibody positivity, B-cell depletion therapy such as intravenous rituximab is an effective treatment.

## Data availability statement

The original contributions presented in this study are included in the article/**Supplementary Material**. Further inquiries can be directed to the corresponding authors.

## Ethics statement

Written informed consent was obtained from the individual(s) for the publication of any potentially identifiable images or data included in this article.

## Author contributions

WL contributed to the conception and design of the study. HL, ZD, RP, and JX collected and organized the clinical data. PH and GY collected and analyzed the kidney biopsy data. WM collected and analyzed the EMG data. SZ collected and analyzed the skin biopsy data. HL and GY drafted the manuscript. All authors contributed to the article and approved the submitted version.

## Funding

This work was supported by a grant to HL from the Science and Technology Development Program of Henan Province (No. 212102310833).

## Acknowledgments

The authors thank their patient for his participation and the EUROIMMUN Weiyi Medical Laboratory for the anti-neuronal antibody tests.

## Conflict of interest

The authors declare that the research was conducted in the absence of any commercial or financial relationships that could be construed as a potential conflict of interest.

## Publisher’s note

All claims expressed in this article are solely those of the authors and do not necessarily represent those of their affiliated organizations, or those of the publisher, the editors and the reviewers. Any product that may be evaluated in this article, or claim that may be made by its manufacturer, is not guaranteed or endorsed by the publisher.

## Supplementary material

Supplementary Video 1 The video shows extensive myokymia in the patient’s lower limbs.

## Abbreviations

MG, myasthenia gravis; PNSs, paraneoplastic syndromes; AChR, acetylcholine receptor; LGI1, leucine-rich glioma-inactivated 1; GABABR, γ-amino-butyric acid-B receptor; ANA, antinuclear antibody; AHA, anti-histone antibody; ECG, electrocardiogram; CT, computed tomography; EMG, electromyography; ANCA, anti-neutrophil cytoplasmic antibody; CASPR2, contactin-associated protein 2; AMPA, alpha-amino-3-hydroxy-5-methyl-4-iso-xazolepropionic acid; aNMT, acquired neuromyotonia.
